# Assessing drug target suitability using TargetMine

**DOI:** 10.12688/f1000research.18214.2

**Published:** 2019-05-28

**Authors:** Yi-An Chen, Erika Yogo, Naoko Kurihara, Tomoshige Ohno, Chihiro Higuchi, Masatomo Rokushima, Kenji Mizuguchi

**Affiliations:** 1 National Institutes of Biomedical Innovation, Health and Nutrition, Ibaraki, Osaka, 5670085, Japan; 2 Shionogi Pharmaceutical Research Center, Shionogi & Co., Ltd., Toyonaka, Osaka, 5610825, Japan

**Keywords:** disease, drug assessment, genetic variation, tractability

## Abstract

In selecting drug target candidates for pharmaceutical research, the linkage to disease and the tractability of the target are two important factors that can ultimately determine the drug efficacy. Several existing resources can provide gene-disease associations, but determining whether such a list of genes are attractive drug targets often requires further information gathering and analysis. In addition, few resources provide the information required to evaluate the tractability of a target. To address these issues, we have updated TargetMine, a data warehouse for assisting target prioritization, by integrating new data sources for gene-disease associations and enhancing functionalities for target assessment. As a data mining platform that integrates a variety of data sources, including protein structures and chemical compounds, TargetMine now offers a powerful and flexible interface for constructing queries to check genetic evidence, tractability and other relevant features for the candidate genes. We demonstrate these features by using several specific examples.

## Introduction

**Figure 1.**
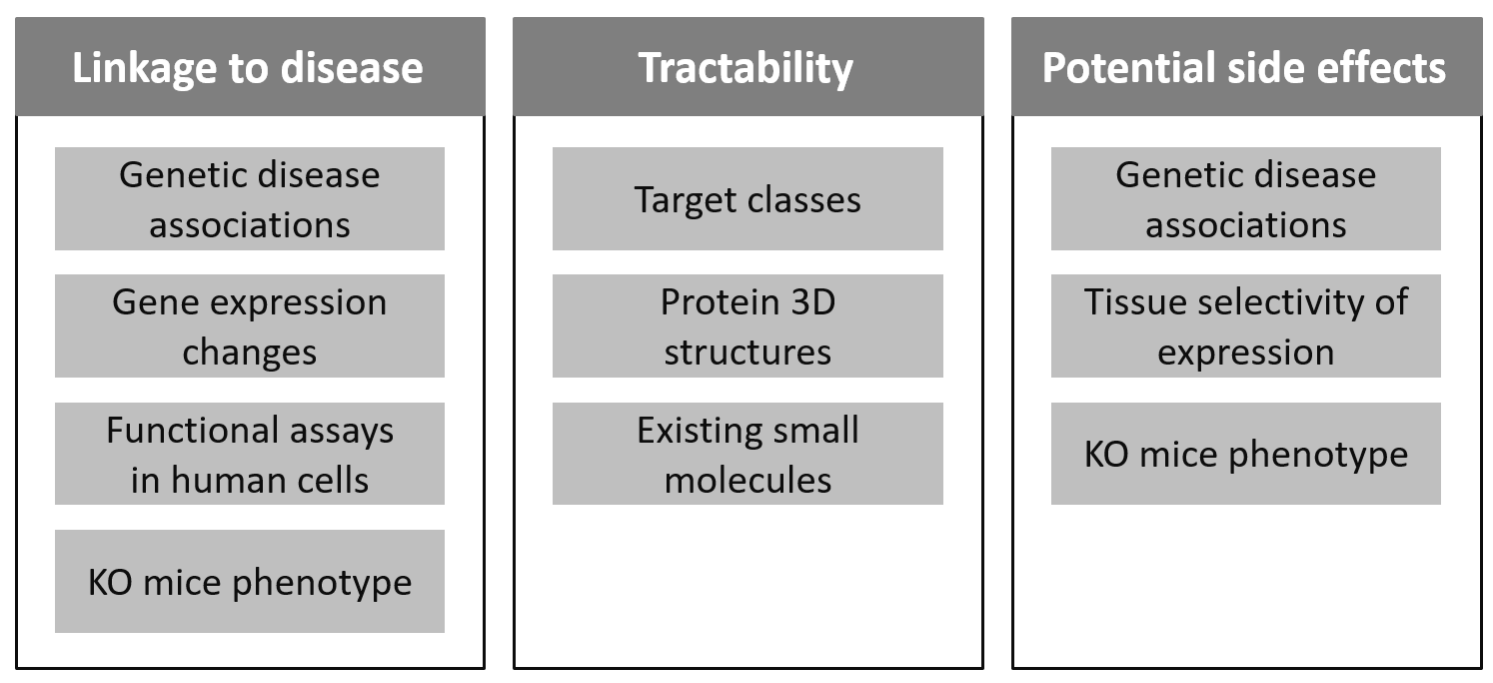
Key factors to be considered in drug target selection. Linkage to disease, tractability and adverse event risk are among the major factors to assess the suitability of novel target candidates. Much of the evidence regarding these factors is available in public domain resources.

A drug discovery project typically begins with the identification of a target molecule. In evaluating potential drug targets, several factors must be taken into account: linkage to disease, tractability (the possibility of finding small molecule compounds with high affinity), potential side effects, novelty, as well as the competitiveness in the market ( Figure 1). Among these factors, the linkage to disease and the tractability are particularly important in terms of the drug efficacy, and become key factors in whether or not the pharmaceutical research and development (R&D) is successful when selecting drug targets ^1, 2^. The most important part of the linkage to disease is genetic associations for the disease or relevant traits. According to analyses reported by AstraZeneca and GlaxoSmithKline, the success rate of such R&D is increased when the choice of the selected target is supported by genetic evidence. The report from AstraZeneca shows that 73% of projects with some genetic linkage of the target to the disease indication in Phase II were active or successful compared to 43% of projects without such data ^3^, while the analysis results from GlaxoSmithKline suggest that selecting genetically supported targets could double the success rate in clinical development ^4^. Several existing resources provide information about genetic evidence, such as DisGeNET ^5^, Open Targets ^6^, and Pharos ^7^. However, a simple list of genes with genetic linkage to the disease is often insufficient for evaluating the disease rationale fully, and additional information and analysis such as pathway enrichment analysis will be needed to assess other aspects of target suitability (e.g. drug mechanisms and safety). In addition, few resources provide tractability information, with the recent update of Open Targets being an exception.

To address these issues, we have updated TargetMine ^8^, a data warehouse for assisting target prioritization, and improved its functionalities for target assessment, particularly in small molecule drug discovery. TargetMine ^8^ utilizes the InterMine framework ^9^ and facilitates flexible query construction spanning a wide range of integrated data sources including those relevant for evaluating linkage to disease and tractability. More specifically, we have integrated new data sources for genetic disease associations including ClinVar, dbSNP, and 1000 Genome Project, incorporated more details of the genome wide association studies from the GWAS catalog, and improved the data model overall to enable more efficient data mining. The new version provides a user-friendly and yet powerful interface to explore the disease rationale for human genes and helps prioritize the candidate genes in terms of both the genetic evidence and target tractability. In addition, with the assistance of InterMine APIs, repeated analysis and queries can be processed efficiently.

## Methods

### Implementation

TargetMine ^8^ is based on the InterMine framework, an open-source data warehouse system designed for biological data integration ^9^. In this update, we added a few customized data sources by defining new data models and implementing new data parsers. Details of how we designed the data models are described in the following sub-sections.

### GWAS catalog

**Figure 2.**
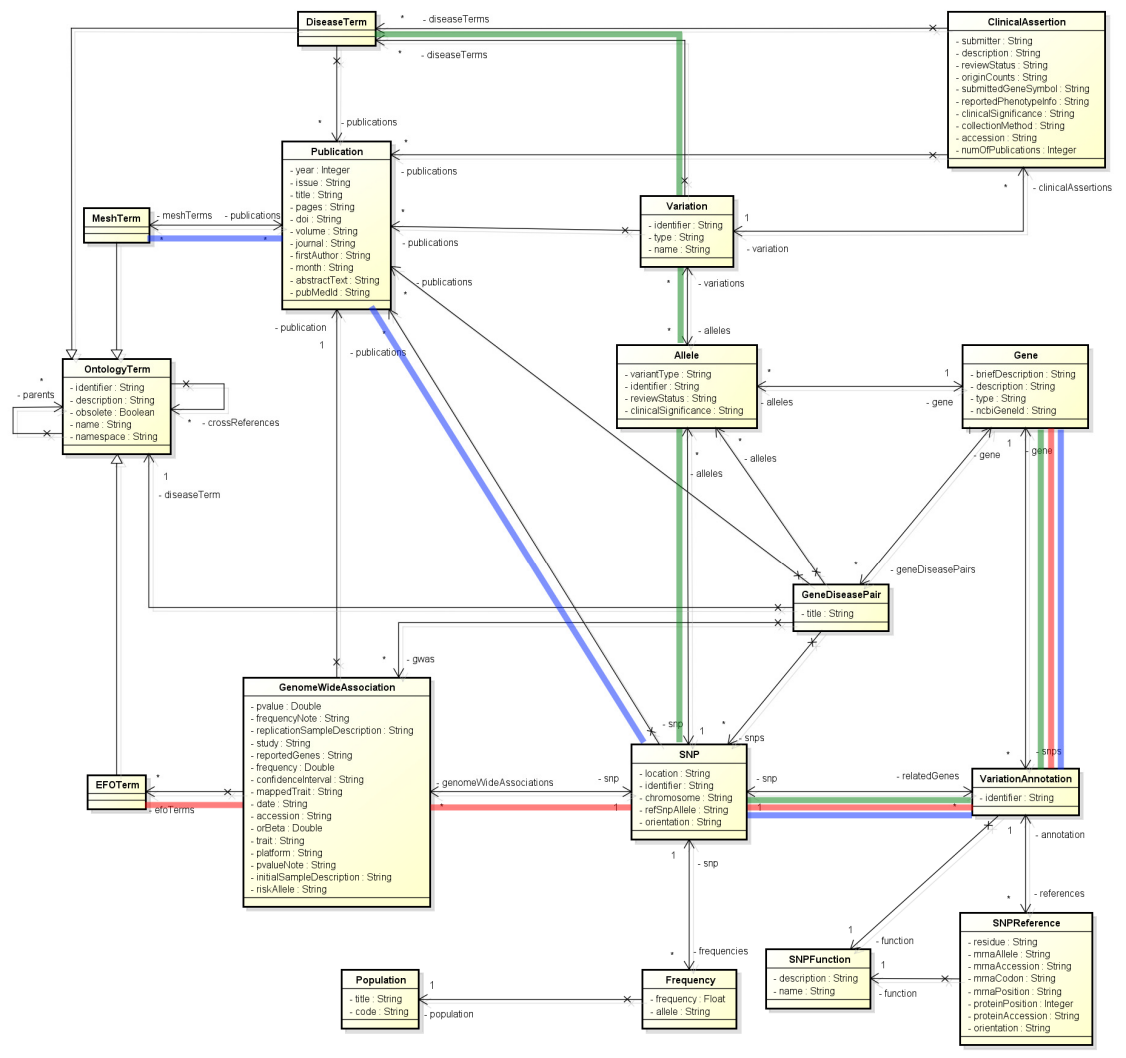
The new implemented data model. The colored lines indicate how the genes and diseases/phenotypes are associated in the post processing step.

The GWAS catalog, founded by NHGRI, is a curated archive of the published genome wide association studies ^10^. We had tried to associate genes to related diseases using the GWAS catalog in the former release of TargetMine ^11^. To annotate disease terms to a trait or study, we first chose the disease ontology (DO) ^12, 13^ and then manually assigned the terms with the assistance of some text matching approaches. However, this process required some knowledge and involved a lot of manual examinations. Thus, it became difficult to keep updating regularly. Fortunately, the curation team started to use experiment factor ontology (EFO) ^14^ to describe the curated GWAS traits in the recent implementation ^15^. EFO covers several domain-specific ontologies that facilitate easier data integration. In our new implemented model, we replace DO terms with EFO terms and also incorporate some more information from each study ( Figure 2). SNP annotations and details of EFO terms are retrieved from the dbSNP database and EFO, respectively.

### ClinVar

ClinVar is a public archive of the relation between human variations and phenotypes ^16, 17^. As defined by ClinVar, a “Variation” could be a single variant, a compound heterozygote, or a complex haplotype. If a haplotype consists of multiple alleles, each allele is assigned with an independent identifier. On the other hand, the same allele could be the member of a different haplotype, thus the relation between the “Variation” and “Allele” is a many-to-many association. An “Allele” is supposed to describe a specific change of a variation, e.g. G>A. However, the SNP entries in dbSNP sometimes merge different combinations of variations (alleles) together if the variations occur at the same genomic position. Thus, an “SNP” entity may contain multiple “Allele” entries in the data model ( Figure 2). Here, we only retrieve the SNP identifier, and the rest of the annotations are integrated from the dbSNP database. The structural variations which reference the dbVar records are not included in the current version. In addition, those alleles which were not assigned with any dbSNP or dbVar identifiers were treated as SNP entities and were stored in TargetMine ^8^ using the information provided by ClinVar. Most of the data were processed from tab delimited files, while some information that were not available in the tab delimited files were processed from XML files. MedGen terms, which are used to integrate the human medical genetic information at NCBI ( https://www.ncbi.nlm.nih.gov/medgen/), were adopted to describe diseases and phenotypes.

### dbSNP

dbSNP is a database which archives short human genetic variations. We first performed a whole data dump to a relational database, and then made queries to extract the necessary information into a flat table. These data include genomic position (based on genome assembly GRCh38), reference mRNA, nucleotide variation, reference protein, and amino acid variation, if available. SNP to gene is a many-to-many relationship, thus we introduce an intermediate class named “VariationAnnotation” to associate them together ( Figure 2). Although the InterMine framework is capable of incorporating whole SNP entries in dbSNP, the integration takes a few days to finish. Considering the frequency that we update TargetMine ^8^ (once a month), it is not very practical to spend a few days doing the integration. As a tradeoff, we decided to store only a subset of SNPs. Only those SNPs which are related with GWAS associations or clinical assertions, or those where there is an associated publication, are selected for storage in TargetMine ^8^.

### Frequency data

Population specific genetic variation frequency is important for evaluating drug efficacy. We preprocessed the frequency data from several data sources, including the Human Genetic Variation Database (HGVD) ^18^, the integrative Japanese Genome Variation Database (1KJPN) ^19^ (download from the archive in National Bioscience Database Center), the Exome Variant Server (EVS) ^20^, and the 1000 Genomes Project (1KGP) ^21, 22^. At the moment, we only incorporate the population specific frequency for those SNPs stored in TargetMine ^8^.

### Post-processing the integrated data

Our implementation allows us to associate the genetic phenotype (disease) and the gene via the GWAS or ClinVar dataset, or moreover the relation that is implied from the disease related MeSH (Medical Subject Headings, https://www.ncbi.nlm.nih.gov/mesh) terms assigned to the correlated publications of the SNPs. In order to make a shortcut and to summarize the available information, we perform post-processing and store the results using a new class named “GeneDiseasePair”. At the moment, there are three types of shortcuts. Gene to SNP to GWAS to EFO terms for GWAS catalog data (the red lines in Figure 2). Gene to SNP to clinical assertions to disease (MedGen) terms (the green lines in Figure 2). And Gene to SNP to publication to MeSH terms (the blue lines in Figure 2). The “GeneDiseasePair” class also includes correlated information including ontology terms, studies, SNPs and publications. These improvements in the data model facilitate quick access from a gene to the associated diseases, annotated by different data sources.

### Operation

TargetMine ^8^ is a Java-based web application that runs on Apache Tomcat. The user interface communicates with the integrated data stored in PostgreSQL, a relational database.

## Use cases

### Querying linkage to disease with TargetMine

To demonstrate the effectiveness of the new version of TargetMine ^8^ in evaluating linkage to disease, we conducted a feasibility study, taking human PCSK9, proprotein convertase subtilisin/kexin type 9, as a typical case. The *PCSK9* gene encodes a protein that promotes degradation of low-density lipoprotein (LDL) receptors in hepatocytes, thereby elevating or maintaining LDL cholesterol levels in the blood. Mutations in this gene are shown to be associated with familial hypercholesterolemia ^23^, and monoclonal antibodies to PCSK9 have been launched on the market as drugs for hypercholesterolemia with and without genetic predispositions ^24, 25^.

**Figure 3.**
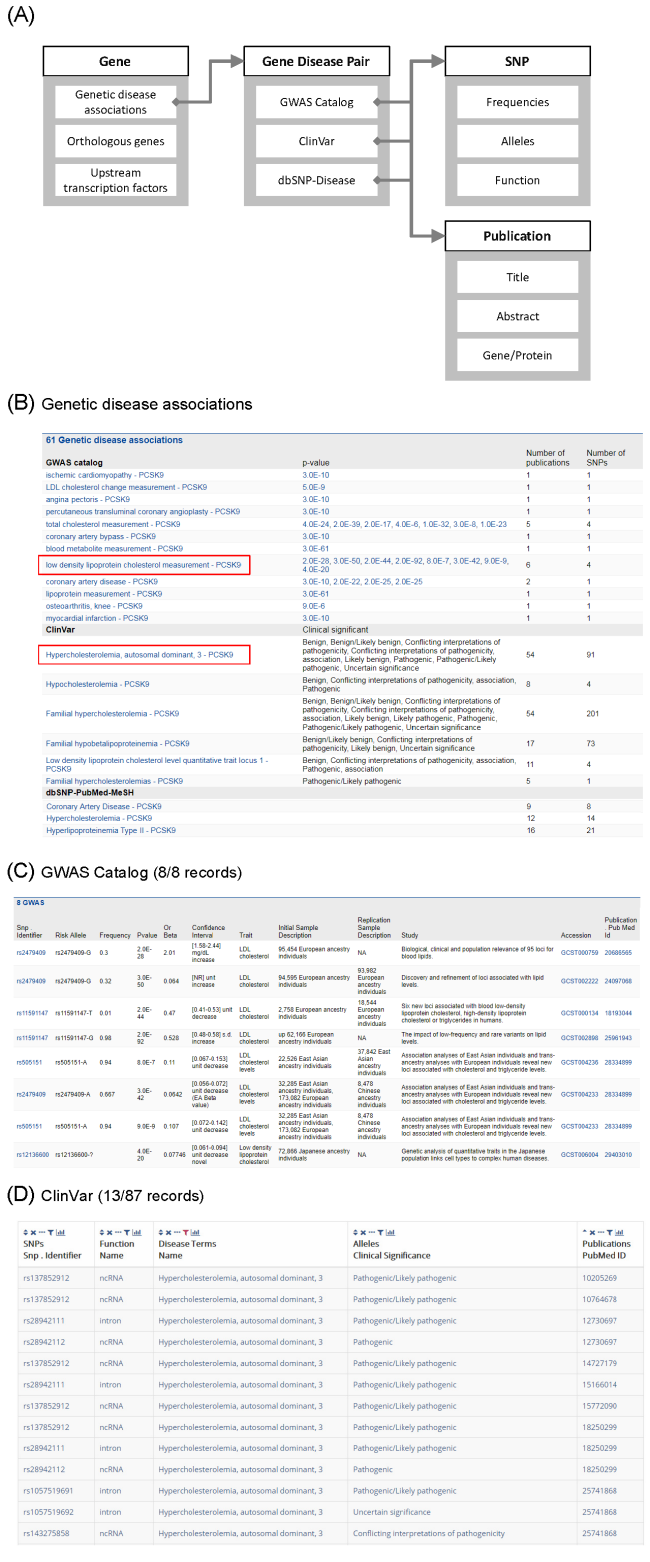
Searching information about linkage to disease with TargetMine. ( **A**) Outline of the procedure for searching. ( **B**) A screenshot of the summary of Genetic disease associations of PCSK9. ( **C**) GWAS records of a pair of PCSK9 and low density lipoprotein cholesterol measurement. ( **D**) ClinVar records of a pair of PCSK9 and hypercholesterolemia, autosomal dominant, 3.

Figure 3A demonstrates a schematic representation of the searching protocol for genetic disease associations with TargetMine ^8^. We first went to a gene report page by searching for the *PCSK9* gene from the top page of TargetMine ^8^ (not shown). From the gene report page, we got information of genetic disease associations ( Figure 3B) as well as many other basic or advanced characteristics such as orthologous genes and upstream transcription factors. The results table of genetic disease associations for *PCSK9* enabled us to confirm that a number of SNPs relevant to this gene have been reported to be associated with plasma LDL cholesterol levels, hypercholesterolemia, or coronary artery disease. By clicking the record of association between “low density lipoprotein cholesterol measurement” and *PCSK9* in the GWAS catalog section ( Figure 3B), we moved to a “gene disease pair” page and checked the details of the GWAS record, including the information on samples, statistical significance and publications ( Figure 3C). Clicking on the SNP identifier (e.g., rs2479409) redirected us to an SNP report page containing the individual SNP basic information (allele, function, literature) and allele frequencies of different human populations (from 1000 Genome Project ^26^ and others, not shown in the figure). Similarly, we examined the associations between “Hypercholesterolemia, autosomal dominant, 3” and *PCSK9* from the ClinVar section in the table ( Figure 3B) and got the details of the ClinVar record such as clinical assertions and publications ( Figure 3D). The publications here reported mutations in *PCSK9* as a cause of autosomal dominant hypercholesterolemia ^23^ (not shown), as mentioned above.

### Querying target tractability for small molecule drugs with TargetMine

We performed another feasibility study to examine whether TargetMine ^8^ provides informative evidence to assess target tractability for small molecules. In this case we also used PCSK9 as an example because no potent small molecule inhibitors for this protein have been reported so far in spite of the intensive research activities of many laboratories ^27^, indicating that PCSK9 is not a highly tractable target.

**Figure 4.**
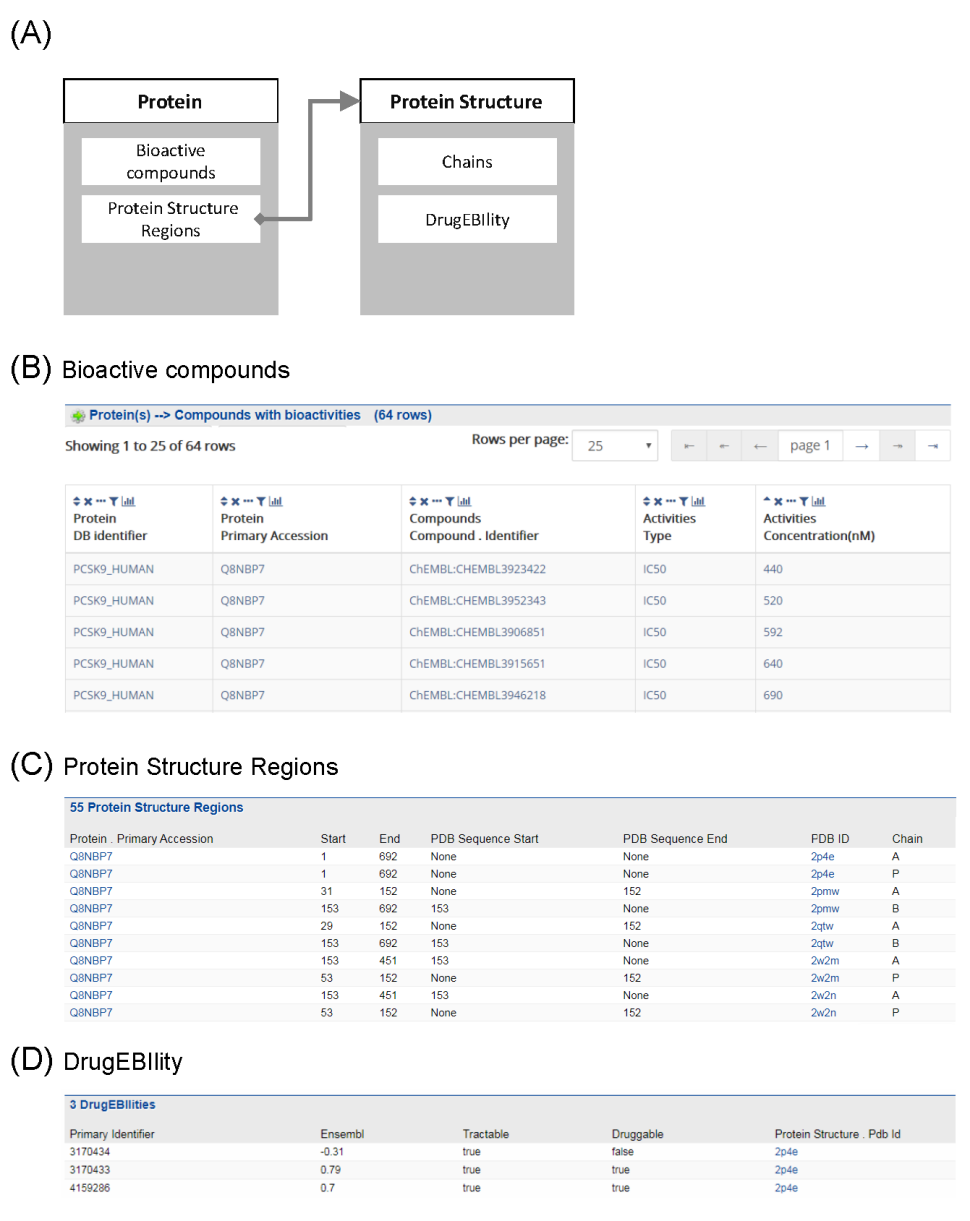
Searching information about target tractability for small molecule drug with TargetMine. ( **A**) Outline of the procedure for searching. ( **B**) Protein structure regions and their ensemble scores calculated by DrugEBIlity. ( **C**) Compounds with bioactivity for PCSK9.

Figure 4A shows a schematic diagram of the procedure of querying tractability with TargetMine ^8^. We first went to the protein report page of PCSK9 and found the bioactive compounds targeting this protein. As we expected, it was revealed that no potent compounds could be found in the ChEMBL database, and the lowest IC50 value was 440 nM (CHEMBL3923422) ( Figure 4B). On the PCSK9 protein report page, we also checked the experimentally determined 3D structures, referred to as “protein structure regions” in TargetMine ^8^, and identified several Protein Data Bank (PDB) entries for this protein ( Figure 4C). Then, we moved to the “Protein Structure” page of a specified PDB ID (2p4e in this case) and found that in the “DrugEBIlity” table (from the DrugEBIlity database), some domains of the PCSK9 protein had positive ensemble scores ( Figure 4D), which are not ligand-based, but structure-based tractability scores. This result indicates that PCSK9 protein may contain some sites/pockets that can bind small molecules, although ensemble scores of DrugEBIlity may need to be further validated.

Collectively, we were able to confirm that the new version of TargetMine ^8^ can quickly provide lines of evidence to assess linkage to disease and target tractability of PCSK9, and that the gathered data correctly reflected the real world situation; namely, it has been a challenge to obtain potent small molecule inhibitors for PCSK9, whereas antibody drugs for this protein have been successfully developed and marketed recently.

### Gathering and prioritizing candidate drug target genes

To assess the utility of the new update of TargetMine ^8^ for prioritizing candidate targets, we conducted a case study where we employed a list of genes associated with hypercholesterolemia in the literature. We tentatively defined three key properties of a novel drug target suitable for small molecules as follows: (1) being associated with hypercholesterolemia via SNPs (GWAS catalog, ClinVar, or dbSNP-Pubmed; see below), (2) having greater than or equal to 50% of protein 3D structures with positive ensemble scores (DrugEBIlity), and (3) having no reported (ChEMBL) potent small molecule inhibitors (IC50 or EC50 ≤ 100 nM), for selecting relatively novel targets. It should be noted that the third criterion here depends on the prioritization purpose; if we attempted to adopt a so-called “me-too” approach, we should select target candidates with potent small molecule inhibitors instead.

**Figure 5.**
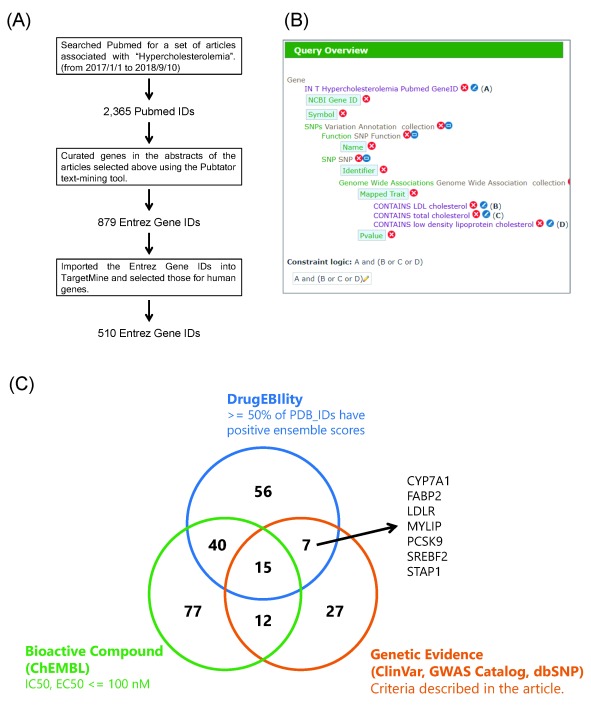
Gathering and prioritizing candidate drug target genes for hypercholesterolemia. ( **A**) Gathering hypercholesterolemia-related genes from article information in PubMed. ( **B**) The screenshot of the query builder in TargetMine to extract the genes with genetic evidence from GWAS catalog. Other query screenshots used in this prioritization process can be found in the extended data. ( **C**) Prioritizing hypercholesterolemia-related genes with TargetMine to identify novel targets for small molecule drugs. Top prioritized genes were defined as those that met all of the following three requirements: 1) more than or equal to 50% of protein 3D structures (PDB IDs) having positive ensemble scores, 2) no potent bioactive compounds (EC50 or IC50 ≤ 100 nM in ChEMBL) and 3) having genetic associations with hypercholesterolemia (for more details, see the Use Cases section).

We first searched PubMed using the term “hypercholesterolemia” (from 2017/1/1 to 2018/9/10) and curated the obtained hits with the “Pubtator” text-mining tool ^28^, resulting in 510 human genes ( Figure 5A). We then selected the genes meeting the requirements defined above using the “Query Builder” in TargetMine ^8^. Figure 5B shows an example of actual query, which aimed to extract the genes with genetic evidence obtained from the GWAS catalog, where “Mapped Trait” contained “LDL cholesterol”, “total cholesterol”, or “low density lipoprotein cholesterol”. Similarly, genes with genetic evidence obtained from ClinVar and dbSNP-Pubmed, with potent small molecules, and those predicted to be tractable in DrugEBIlity database were also extracted using the query builder. (The relevant data are shared as the supplementary materials on the TargetMine website; see “Data Availability” for the URL.) Thus, the new implementation enabled us to filter objects on complex conditions with a user-friendly, intuitive graphical interface.

Genes that satisfied all three requisites above are presented in Figure 5C ( *CYP7A1*, *FABP2*, *LDLR*, *MYLIP*, *PCSK9*, *SREBF2* and *STAP1*). Among the seven genes we found *MYLIP* and *STAP1*. MYLIP is an E3-ubiquitin ligase that degrades LDL receptors in the liver, which are therefore considered to be a potential therapeutic target for dyslipidemia ^29^. Similarly, the *STAP1* gene has been recently annotated as a fourth locus associated with autosomal-dominant hypercholesterolemia, and might be a novel target for therapeutic development of hypercholesterolemia ^30^. This result suggests that the new version of TargetMine ^8^ allows us to effectively prioritize target candidate genes in terms of linkage to disease, tractability and competitors. On the other hand, the list includes intractable targets such as PCSK9 and LDLR, indicating the need for improvement of the data and/or the thresholds with which tractable proteins are selected (in this study, ≥50% of protein 3D structures have positive ensemble scores in DrugEBIlity database).

### Evaluating candidate drug target genes in the context of pathways

**Figure 6.**
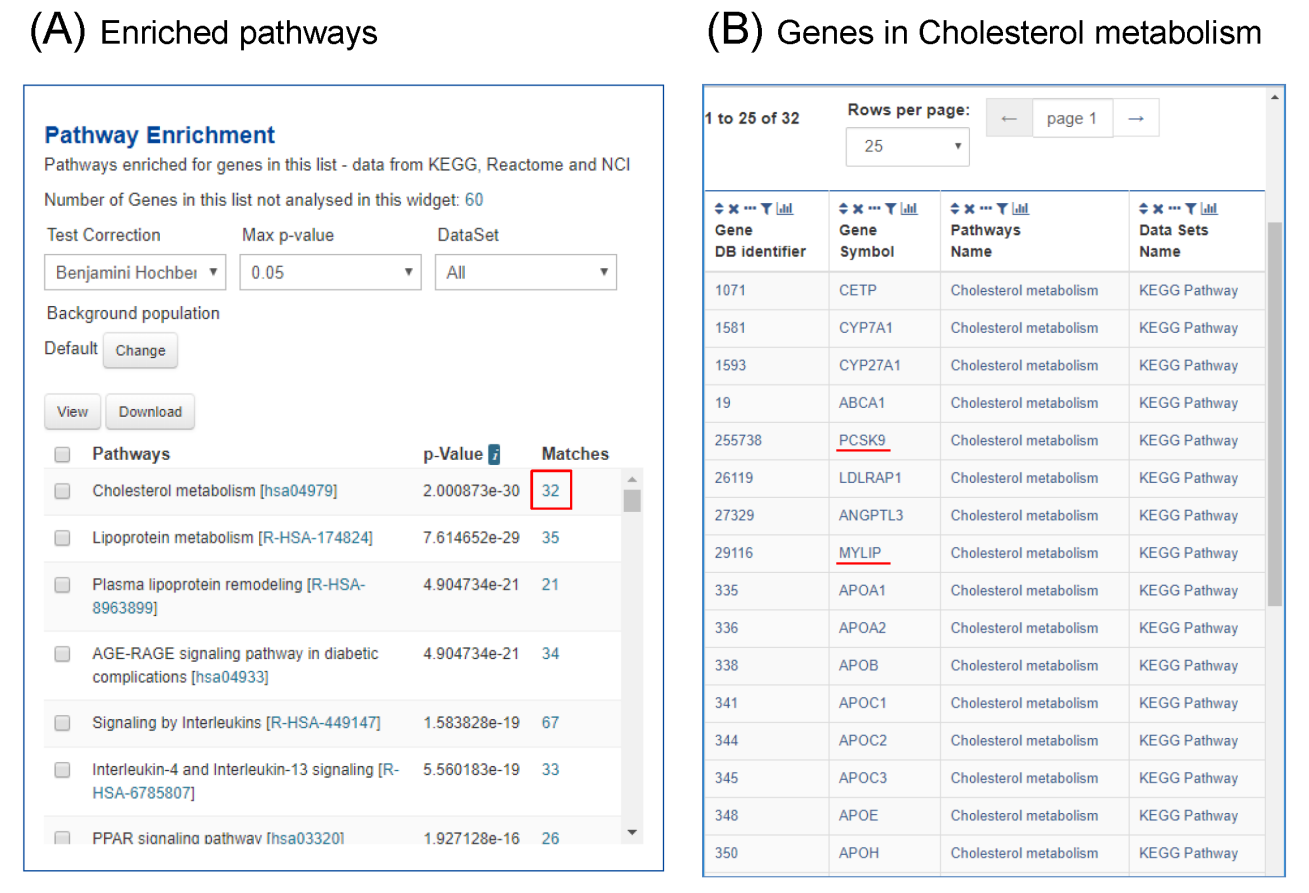
An example of pathway enrichment analysis. ( **A**) Result of pathway enrichment analysis of the 510 human genes, which were described in Figure 5A. ( **B**) Genes overlapped between the 510 human genes and those included in “Cholesterol metabolism” pathway.

TargetMine also allows us to seamlessly conduct pathway analysis, which identifies biological processes or pathways that are statistically enriched in a list of genes. From the perspective of prioritizing target candidates, pathway analysis is useful for highlighting the genes on pathways targeted by existing drugs or pathways with concerns for adverse events when blocked. Figure 6 shows the result of pathway enrichment analysis of the 510 human genes, which were obtained by PubMed search in Figure 5A using the term “hypercholesterolemia”. As expected, the most highly enriched pathway is “Cholesterol metabolism”, followed by “Lipoprotein metabolism”. It is also readily recognizable that PCSK9 and MYLIP (mentioned above), both of which are involved in the degradation of LDL receptors, are on the same pathway (i.e., cholesterol metabolism) ( Figure 6B). This observation may suggest that discovering MYLIP inhibitors should not be so attractive because antibody drugs for PCSK9 have already been marketed, although small molecules against MYLIP may still have some advantages, such as lower cost and better compliance (oral availability), against anti-PCSK9 antibodies.

## Conclusions

These use cases demonstrate that the updated version of TargetMine ^8^ can be applied in pharmaceutical R&D, from the aspect of understanding the linkage to disease, examining the tractability of targets and prioritizing candidates. The recent update of the Open Targets platform ^31^ also starts to cover “DrugEBIlity” data and protein structural information, suggesting that an integrated resource containing gene-disease associations and tractability information is indispensable for the pharmaceutical R&D. In addition, taking advantage of the features of the InterMine framework, TargetMine ^8^ also facilitates more flexible and more complex queries for advanced users.

## Data availability

### Underlying data

The TargetMine data warehouse is publicly available at https://targetmine.mizuguchilab.org.

### Extended data

Open Science Framework: Hypercholesterolemia related genes and TargetMine queries. https://doi.org/10.17605/OSF.IO/FUW53 ^32^

This project contains the following extended data:

- Hypercholesterolemia_PubMed_gene_list.tsv (The list of the 510 genes used in the use case, described in Figure 5A.)
- q_Hypercholesterolemia_ClinVar.xml (The query for extracting the genes with genetic evidence from ClinVar.)
- q_Hypercholesterolemia_dbSNP_PubMed.xml (The query for extracting the genes with genetic evidence from the associated publications in dbSNP.)
- q_Hypercholesterolemia_Bioactive_Compound.xml (The query for retrieving the genes with bioactive compounds.)
- q_Hypercholesterolemia_DrugEBIlity.xml (The query for retrieving genes with the DrugEBIlity scores.)
- q_Hypercholesterolemia_GWAScatalog.xml (The query for extracting the genes with genetic evidence from GWAS catalog.)

Extended data are available under the terms of the Creative Commons Zero “No rights reserved” data waiver (CC0 1.0 Public domain dedication).

## Software availability

Source code available from: https://github.com/chenyian-nibio/targetmine

Archived source code at time of publication: https://doi.org/10.5281/zenodo.2573565 ^8^.

License: MIT License.
