# Supplementary material for: Assessing drug target suitability using TargetMine
Source: F1000Res. 2019 May 28;8:233. Originally published 2019 Feb 28. [Version 2] doi: 10.12688/f1000research.18214.2 (PMC6439796; doi:10.12688/f1000research.18214.2)
Supplement: Peer review history

# Peer review: Reviewer response for version 1

**DOI:** 10.5256/f1000research.19924.r45061

**Published:** 2019-03-28

**Authors:** Anne Hersey

**Affiliations:** 1 European Bioinformatics Institute (EMBL-EBI), Cambridge, UK

The authors have produced a nice tool (TargetMine) that can be used for prioritizing targets for drug discovery. They have done this by integrating data from a range of other resources that have the potential to identify the relevance of targets for diseases of interest as well as the likelihood that a small molecule can be found for that target (tractability). The authors have also provided all the code in the form of a github repository and the data model for TargetMine is shown in the article.  

 They describe adequately how to use the resource with the aid of a couple of specific use cases that they work through in the article. I have followed through the first example and can obtain the same results as they do. The 2 ^nd ^example starts from a series of PubMed articles so is less easy to reproduce oneself although the logic is sensible. TargetMine also has links to tutorials that can be used to guide a user through using the resource although I haven’t tried these. In the introduction the authors mention not just using genetic data to assess the relevance of targets to disease but additional information such as pathway analysis although this isn’t exemplified in their use cases; perhaps their use cases could be expanded to include this? In the 2 ^nd ^use case it would be good to describe at the start why they were prioritizing targets which had no reported inhibitors with IC50s <100nM. I assume this was because they were looking for novelty but this is not explained. To me the 15 targets at the centre of the Venn diagram (figure 5) would also be relevant and tractable targets.

 I think it would be useful to be able to link back to the source databases that the information comes from. For example, when I select a UniProt ID I would expect to link back to UniProt itself. Likewise, it would be good to be able to link back to PDB where you can view the 3D structure of the protein. I couldn’t find a way of doing this.  

 As the authors mention in their conclusion the Open Targets Platform has also recently started to use the DrugEBIlity algorithms. The authors might be interested to know that this is not currently being further updated and will soon be replaced with an alternative tractability prediction method that will also be free and openly available. A minor but important point: the authors use the words “Ensembl” and “ensemble” to refer to the ensemble of prediction models that are used as one of the measures of tractability. “Ensemble” is the correct word which is very different from “Ensembl” the genome browser ( https://www.ensembl.org/). I think it would be useful if the authors could correct this throughout the article.

I have read this submission. I believe that I have an appropriate level of expertise to confirm that it is of an acceptable scientific standard.

# Peer review: Reviewer response for version 1

**DOI:** 10.5256/f1000research.19924.r45533

**Published:** 2019-03-27

**Authors:** Rachel Lyne

**Affiliations:** 1 Department of Genetics, University of Cambridge, Cambridge, UK

The authors describe data updates to the already established TargetMine database. The updates allow candidate genes to be checked for genetic evidence for disease and target tractability. The authors clearly demonstrate the utility in integrating the new data through a set of use cases. The sophisticated web interface allows for powerful exploration and advanced querying of the data, making the resource a valuable tool for pharmaceutical research and development. The following minor points should be addressed:

1. In the introduction it is mentioned that additional information and analysis, such as pathway enrichment, are needed to assess aspects of target suitability, but such analysis is not shown in the use-cases. It would be useful to demonstrate features that TargetMine provides that are not available in similar systems such as Open Targets and Pharos. In addition the authors describe how the new data can be used but do not tie this in with the wealth of data already available in TargetMine which could also be used for tractability studies, such as uniprot and interpro protein domains.
2. TargetMine includes an extensive API but this is not mentioned in the paper. Access to this data through an API could have clear advantages for anyone wishing to set up a workflow or provide a more automated analysis.
3. The use case “Gathering and prioritising candidate drug target genes” would be difficult for someone unfamiliar with searching an interMine-based database to reproduce. First, the set of genes (or the filtered set of human genes) should be provided as supplemental material. An overview of a set of queries constructed using the TargetMine query builder is provided. However, to reproduce this set of queries using the query builder is not immediately obvious for a naive user. A more detailed set of screenshots as supplementary material could help, or provision of the set of queries as a series of template searches.
4. A note on how often the data will be updated should be included.
5. Plans for further data additions could be included.
6. The reference for TargetMine (8) should be updated to contain the correct author list.

I have read this submission. I believe that I have an appropriate level of expertise to confirm that it is of an acceptable scientific standard.
